# Consumption of fruit and vegetables among elderly people: a cross sectional study from Iran

**DOI:** 10.1186/1475-2891-9-2

**Published:** 2010-01-13

**Authors:** Leili Salehi, Hassan Eftekhar, Kazem Mohammad, Sedigheh Sadat Tavafian, Abolghasem Jazayery, Ali Montazeri

**Affiliations:** 1 Department of Health Education, School of Public Health, Tehran University of Medical Sciences, Tehran, Iran; 2 Department of Epidemiology and Statistics, School of Public Health, Tehran University of Medical Sciences, Tehran, Iran; 3 Department of Health Education, Faculty of Medicine, Tarbiat Modares University, Tehran, Iran; 4 Department of Nutrition, School of Public Health, Tehran University of Medical Sciences, Tehran, Iran; 5 Department of Mental Health, Iranian Institute for Health Sciences Research, ACECR, Tehran, Iran

## Abstract

**Background:**

There is substantial evidence that low consumption of fruit and vegetables (FV) is a major risk factor for many chronic diseases. The aim of this study was to assess FV consumption and the variables that influence it among elderly individuals in Iran aged 60 and over.

**Methods:**

This was a cross-sectional study to investigate FV intake by a randomly-selected sample of members of elderly centers in Tehran, Iran. A multidimensional questionnaire was used to collect data on demographic characteristics, daily consumption of FV, knowledge, self-efficacy, social support, perceived benefits, and barriers against FV. Data were analyzed using t-tests, one way analysis of variance, Pearson correlation, and logistic regression.

**Results:**

In total, 400 elderly individuals took part in the study. The mean age of the participants was 64.07 (SD = 4.49) years, and most were female (74.5%). The mean number of FV servings per day was 1.76 (SD = 1.15). Ninety-seven percent of participants (n = 388) did not know the recommended intake was at least five servings of FV per day. Similarly, 88.3% (n = 353) did not know the size of a single serving. The most frequent perceived benefits of and barriers against FV consumption were availability and expense, respectively. Knowledge (OR = 0.59, 95% CI = 0.39-0.88), perceived benefits (OR = 0.92, 95% CI = 0.88-0.96) and barriers (OR = 1.08, 95% CI = 1.04-1.14), self-efficacy (OR = 0.89, 95% CI = 0.83-0.95) and family support (OR = 0.91, 95% CI = 0.83-0.99) were significantly associated with fruit and vegetable consumption.

**Conclusion:**

The findings of this study indicate that FV intake among elderly individuals in Iran was lower than the recommended minimum of five daily servings and varied greatly with age, marital status, educational attainment, and income level. The results also indicated that low perceived benefits, low self-efficacy, and perceived barriers could lead to lower consumption of FV. It seems that in order to improve FV consumption among elderly individuals in Iran, raising awareness, improving perception of benefits and enhancing self-efficacy regarding FV consumption should receive more attention. Indeed, it is essential to plan health education programs and nutritional interventions for this group of the population.

## Background

Coronary heart disease (CHD), cancer and stroke are leading causes of death [1] that are more prevalent among elderly individuals [2] and there is substantial evidence that low intake of fruit and vegetables (FV) is a major risk factor for such diseases [3]. Several studies have shown that adequate consumption of fruit and vegetables is associated with a reduced risk of cancer [4] and CHD [5]. Furthermore, previous studies have shown strong negative relationships between FV intake and obesity [6], diabetes [7] and hypertension [8]. Despite all these benefits, people do not properly follow the minimum recommended consumption of five servings of FV per day [9]. Data on FV intake derived from food balance sheets in 21 countries (mainly developing countries) showed that only in three of those countries did FV intake meet the minimum World Health Organization (WHO) recommended consumption [10], although the use of food balance sheets as a single source of information may have weaknesses [11].

In general, many factors contribute to FV intake. For instance, one study showed that knowledge, attitudes, skills and self-efficacy were among the factors that might influence an individual's likelihood of taking five servings of FV per day [12]. It has also been shown that promoting healthy eating behaviors would be successful if influencing factors were considered through appropriate models of health behavior change [13]. One of the most popular models for studying behavioral change in health education/promotion is the Transtheoretical Model (TTM). This assumes that health behavior change involves progress through six stages: pre-contemplation, contemplation, preparation, action, maintenance, and termination [14,15]. Studies have demonstrated that the TTM is effective in predicting and promoting fruit and vegetable consumption in different population groups [16,17].

Many studies have revealed variables that influence FV consumption among elderly populations worldwide [18,19], but little is known about the frequency, distribution, and determinants of FV consumption among elderly individuals in Iran [20]. The only study that has reported the exact amount of FV consumption among the elderly in Iran was an investigation among a Middle Eastern elderly population including Iranians. It reported that the average FV intake was 358 g/day in males and 349 g/day in females [21]. In Iran the total mean energy intake exceeded requirements and was mainly obtained from carbohydrates such as simple sugar. The Iranian diet is mainly composed of bread and rice as major energy sources, while chronic diseases are the main causes of mortality and morbidity in Iran [22] and their trend in the country is rising [23,24]. In Iran, cardiovascular disease (CVD) accounts for 38% of deaths [25]; the second most common cause of death is road traffic-related injuries [26], and the third is cancer [27]. Diabetes accounts for 7.7% [28], and 82% of women and 63% of men aged 50 years and above are overweight [29]. It seems possible that this profile of the country's health status could be improved in part if Iranians, especially those in higher risk groups such as elderly individuals, took adequate amounts of fruit and vegetables.

The present study was conducted to investigate factors that contribute to FV consumption by elderly individuals in Iran. We were particularly interested in studying the effects of the following determinants: age, gender, education, marital status, employment and economic status, chronic disease, body mass index (BMI), stages of change, self efficacy, support from family and friends, knowledge regarding FV, and perceived benefits and barriers.

## Methods

### Design and data collection

This was a cross-sectional study among a randomly selected sample of members of elderly centers in Tehran, Iran. The study was conducted between September 2007 and April 2008. Tehran has 23 elderly centers in which free educational and social services are offered to members. Membership is free of charge and all individuals aged 60 and above are eligible to join. A random sample was selected from all listed members. The sample size was estimated on the basis of a single proportion design. We assumed that at best 50% of the elderly would have adequate FV intake. Thus, a study with a sample of 385 elderly would have 80% power to detect a difference of 5% (45-55%) at the 0.05 significance level. The sample size actually obtained for this study was 454. Those who did not agree to take part in the study and those who were suffering from serious illness or had undergone a surgical operation up to 3 months before the date of data collection were excluded (n = 422).

The Ethics Committee of Tehran University of Medical Sciences approved the study. Before the study was conducted, the aim, method and confidentiality were explained fully to the potential participants and if they were satisfied and willing to take part they were asked to read and sign a consent form. The headmasters of all 23 elderly centers were also approached about their willingness to participate. To collect data, trained interviewers conducted face-to-face interviews. The interviewers had high school diplomas. Each interview lasted for approximately 45 minutes.

### Instruments

We used several instruments to collect data. Their reliability was assessed among a sub-sample of 20 participants using Cronbach's alpha coefficient. The instruments and findings are explained as follows:

#### 1. Demographic and anthropometrics questionnaire

This comprised three sections covering demographic and anthropometrics data including information on age, sex, education, income, marital status, health status (having a chronic disease or not) and BMI. Chronic disease was indicated by asking each individual to respond to the question: 'Do you have any long-standing diseases'? Anyone who responded positively was asked to indicate the name of the disease. Weight was measured using the same digital scales (SECA, calibrated in Iran) while the subjects were minimally clothed and not wearing shoes. Height was measured by a tape measure while the subjects were standing and not wearing shoes and the shoulders were in a normal position. BMI was calculated and expressed in kg/m^2^, and economic status was measured using the asset-based approach developed by Ferguson and colleagues [30] and used in previous cross-national studies of economic status and health in developing countries [31]. According to this scale, 0-3 assets were considered low, 4-6 were considered intermediate and 8 or more were considered high economic status. The items considered as assets were: television, refrigerator, washing machine, microwave oven, dishwasher, computer, electrical sweeper, automobile and phone.

#### 2. Stages of change questionnaire regarding FV consumption behavior

This part of the questionnaire comprised five statements by which the participants were categorized into different stages of change: pre-contemplation, contemplation, preparation, action and maintenance. This part included multiple-choice questions adapted from the literature [32]. The participants were asked to choose the statement that best described their status. Choices for the questions were (1) I am not currently consuming five servings of FV a day and I am not thinking of doing so in the upcoming six months, (2) I am not currently consuming five servings of FV a day but I plan to do so within the next six months, (3) I am not currently consuming five servings of FV a day but I plan to do so within the next month, (4) I am currently consuming five servings of FV a day but I have been doing so for less than six months, and (5) I am currently consuming five servings of FV a day and I have been doing that for more than six months. The internal consistency of the questionnaire was assessed using Cronbach's alpha coefficient and found to be 0.79.

#### 3. Self-efficacy rating scale

Self-efficacy was assessed using a five-item questionnaire developed by Ma et al. [32]. Each item is rated on a 5-point scale (from not at all confident to very confident about recommended FV consumption) and the questionnaire gives a score ranging from 5 to 25. A higher score indicates a greater degree of self-efficacy. The scale showed good validity (as assessed by content validity) and satisfactory internal consistency (as measured using Cronbach's alpha coefficient, 0.85).

#### 4. Support rating scale

In order to assess the influences of family and friends on healthy eating, the 6-item family support for healthy eating habits scale and the 6-item friend support for healthy eating habits scale were used [33]. Each item is rated on a 5-point scale (from none to very often) and the overall score ranges from 6 to 30 for each section. The Cronbach's alpha coefficient for the scale was 0.85.

#### 5. Knowledge instrument regarding FV consumption

The knowledge of participants was measured using a six-item questionnaire. The first question was: Would you say what is the recommended number of servings for FV consumption per day?" The response categories were '1', '2-3' and '5 or more'. The second item was: Would you say that a single serving of beans is 'more,' 'less,' or 'about as much' as can fit in the palm of your hand? The next four items used an agree/disagree response format. The items were: FV are a good source of fiber; if you take vitamin pills you do not have to eat a lot of FV; boiling and evaporation is the best method to cook vegetables; as long as you eat FV, it does not matter what color they are. Correct responses were summed to create a total knowledge score of 0 to 6. This scale was found to be valid (approved by ten nutrition specialists) and a reliable measure (Cronbach's alpha coefficient, 0.72).

#### 6. Perceived benefits and barriers regarding FV consumption

This part was generated from previous studies and focus group discussions with convenient samples of elderly individuals. Participants were asked about their perceptions regarding amounts of FV intake. The final perceived benefit questionnaire consists of 15 items. Each item is rated on a 5-point scale ranging from very important to not at all important. The perceived barrier consists of 11 items. Each item is also rated on a 5-point scale ranging from very important to not at all important. The total score for the perceived benefits ranges from 15 to 75 and for perceived barriers from 11 to 55. The Cronbach's alpha coefficient for the benefit scale was 0.73 and for the barrier scale it was 0.69.

#### 7. Daily FV consumption

This section comprised two parts as follows:

**7.1. Food frequency questionnaire: ** This consisted of two main questions related to fruits and vegetables (38 items in all) available in Tehran's markets. Response categories were: never, 1-2 times per week, 3-4 times per week, 5-6 times per week, and every day. Accordingly the respondents were asked to indicate the amount of intake. We estimated the daily FV intake for each individual on the basis of this information.

**7.2. A 24-hour recall:**  Participants were asked to estimate their daily servings of FV at breakfast, lunch, dinner, and between meals as snacks or deserts in accordance with a nutrition guideline card. The nutrition guideline card categorized one serving of vegetables into one of three following groups: (1) one cup of raw green leafy vegetables such as spinach or salad; (2) one-half cup of other vegetables cooked or chopped raw, such as tomatoes, carrots, pumpkin, corn, Chinese cabbage, beans, or onions; and (3) one-half cup of vegetable juice. The nutrition guideline categorized one serving of fruit into one of three groups: (1) one medium size fruit such as an apple, banana, or orange; (2) one-half cup of cooked, chopped, or canned fruit; and (3) one-half cup of fruit juice, not artificially flavored. We then calculated daily serving FV consumption for each individual.

### Data analysis

Data were analyzed using both descriptive and analytic statistics. Mean values of daily FV consumption were provided by the characteristics of the study sample; and for knowledge, benefits, barriers, and self-efficacy, mean values were assessed by stages of change.

To compare FV consumption per day among different subgroups of the study sample, an independent samples t-test and one-way analysis of variance (ANOVA) were used to compare two or more independent mean values, respectively.

Pearson correlation was used to assess the correlation between independent variables and FV consumption.

Logistic regression was used to identify the magnitude of association between FV servings eaten per day and independent variables including age, gender, education, marital status, economic status, self-reported chronic disease, perceived benefits and barriers, self efficacy, knowledge regarding FV consumption, and social support (family and friends). To avoid infinite odds ratios, some categories were merged. For example, marital status was categorized into 'married' and 'widowed and divorced'. For the purpose of logistic regression analysis the sample was divided into quartiles of FV consumption and the 1st (inadequate) and 4th (adequate) quartiles were compared in order to increase statistical power.

## Results

Of 454 eligible individuals, 32 did not agree to be interviewed. Thus, 422 individuals who signed the consent form entered the study. A total of 22 questionnaires were excluded from analysis owing to incomplete answers. In total, 400 elderly individuals (102 men and 298 women) from 23 elderly centers took part in the study. The mean age of participants was 64.07 (SD = 4.49) years (range 60-87). Most participants were married (55%) and unemployed (80%) with BMI between 25 and 29 (48%). The results showed that FV consumption among the participants was low. Overall, the mean serving of FV intake eaten per day for the whole sample was 1.76 (SD = 1.15) and varied significantly with age (P = 0.003), educational attainment (P < 0.001), marital status (P < 0.001), economic status (P < 0.001), and BMI (P < 0.001). However, there were no significant differences between males and females or with different employment status. Table 1 shows the characteristics of the study sample and the mean servings of FV per day for the study subgroups.

**Table 1:** The characteristics of study sample (n = 400)

	No (%)	FV serving/day Mean (SD)	P*
**Age**			0.003

60-64	255 (63.8)	1.83 (1.18)	

65-69	87 (21.7)	1.73 (1.10)	

70-74	47 (11.75)	1.52 (1.04)	

75-79	8 (2)	1.53 (0.88)	

80-84	2 (0.5)	0.57 (0.61)	

>85	1 (0.25)	0.29 (0.29)	

**Gender**			0.81

Male	102 (25.5)	1.74 (1.16)	

Female	298 (74.5)	1.77 (1.50)	

**Education**			< 0.001

Illiterate	165 (41.2)	1.63 (0.96)	

Primary	143 (35.8)	1.74 (1.15)	

Junior secondary	64 (17)	1.57 (0.98)	

Senior secondary	22 (5.5)	3.15 (1.73)	

College	6.0 (1.5)	2.98 (1.54)	

**Marital status**			< 0.001

Married	230(55)	1.95(1.16)	

Widowed/divorced	170 (45)	1.51(1.08)	

**Economic Status**			< 0.001

Low (0-3 assets)	306 (76.5)	1.68 (1.03)	

Intermediate (4-6 assets)	65 (16.3)	1.57 (0.99)	

High (8 or more assets)	29 (7.3)	3.34 (1.52)	

**Employment status**			0.58

Employed	54 (13.5)	1.91 (1.04)	

Housewife	283 (70.8)	1.73 (1.11)	

Retired	63 (15.7)	1.79 (1.41)	

**BMI**			< 0.001

<25	106 (26.5)	2.78 (1.15)	

25-29	192 (48)	1.66 (0.74)	

≥ 30	103 (25.5)	0.92 (1.01)	

**Stage of change (n = 386)**			< 0.001

Precontemplation	283(73.4)	1.63 (0.98)	

Contemplation	76 (19.6)	1.47 (0.94)	

Preparation	27 (7.00)	2.28 (0.68)	

Action	6.0 (1.50)	5.0 (0.00)	

Maintenance	8.0 (2)	5.25 (0.70)	

**Chronic disease**			< 0.001

Yes	197 (49.25)	2.03 (1.29)	

No	203 (50.75)	1.49 (0.92)	

* Derived from independent samples t-test and one was analysis of variance (ANOVA).

The data analysis indicated that 97% of participants (n = 388) did not know that the recommended intake is at least five servings of fruit and vegetables per day. Similarly, 93% (n = 372) did not know about the importance of FV color, and 88.3% (n = 353) did not know the recommended size of one serving. However, 74.3% of participants (n = 297) acknowledged that FV are an important source of fiber, 70.3% (n = 281) correctly reported that vitamin pills were not as valuable as FV, and 76% (n = 304) were aware that boiling and evaporating are healthy methods of cooking vegetables. The mean scores of participants' knowledge were 2.07 (SD = 0.47), 2.91 (SD = 0.84), 3.15 (SD = 0.82), 4.33 (SD = 0.52) and 5.75 (SD = 0.46) for the pre-contemplation, contemplation, preparation, action and maintenance stages, respectively.

The perceived benefits and barriers regarding FV intake are shown in table 2. The most frequent perceived benefits and barriers towards FV consumption were availability (95%) and expense (55.5%), respectively. The mean scores for the perceived benefits were 55.04 (SD = 8.89) for pre-contemplation, 55.80 (SD = 8.19) for contemplation, 58.30 (SD = 6.99) for preparation, 62.50 (SD = 7.5) for action, and 67.50 (SD = 10.57) for maintenance. The mean scores for the perceived barriers were 35.62 (SD = 8.17) for pre-contemplation, 35.05 (SD = 6.57) for contemplation, 32.41 (SD = 8.39) for preparation, 27.50 (SD = 5.68) for action, and 27.88 (SD = 15.79) for maintenance. The mean scores for self-efficacy were 12.65 (SD = 6.24), 12.92 (SD = 6.45), 15.67 (SD = 5.82), 17.0 (SD = 4.14) and 17.41 (SD = 3.59) for pre-contemplation, contemplation, preparation, action and maintenance, respectively.

**Table 2:** Frequency of responses to survey questions regarding the benefits and barriers of FV consumption

Perceived benefits		**No**.	%
	I can find any kind of FV in my local stores	381	95.25

	FV contain more vitamins and minerals	379	94.75

	FV decrease the risk of chronic disease	330	82.50

	FV make our diet diverse	327	81.75

	Eating FV is a good way for treating chronic disease	330	82.50

	Eating FV would help me to be less aggressive	317	79.25

	Eating FV treats constipation	315	78.75

	Eating FV would help me maintain my weight	311	77.75

	Eating more FV advised by physicians	285	71.25

	Eating FV cheering my family members	274	61.75

	Eating FV is common in my culture	264	66.00

	Eating FV would keep me from getting sick	145	36.25

	Eating FV would help me to live longer	130	32.50

	I feel I am caring my body health if I eat more FV	119	29.75

	By eating FV I feel better	59	14.75

**Perceived barriers**			

	Eating FV is expensive	222	55.50

	Habit of eating FV has been established since childhood	153	38.25

	Eating FV leads to overeating	147	36.75

	Media advertisements are not about eating FV	127	31.75

	I do not have time to prepare FV	107	26.75

	Eating more FV is not recommended in my culture	95	23.75

	My family members do not like consumption of FV	82	20.50

	Eating more FV is difficult for me	82	20.50

	I have health problems (like flatus) with eat FV	80	20.00

	I have limitation ways to provide FV in my meal	80	20.00

	I do not like taste of FV	38	9.50

TTM variables, knowledge, social support and FV intake were all found to be significantly correlated (Table 3).

**Table 3:** Correlation between TTM, knowledge, social support and FV consumption

	FV intake	Knowledge	Benefits	Barriers	Self efficacy	Social support (family)	Social support (friends)
FV intake	1						

Knowledge	0.39**	1					

Benefits	0.23**	0.13**	1				

Barriers	-0.29	-0.20	-0.03	1			

Self efficacy	0.33**	0.16**	0.09	-0.17**	1		

Social support (family)	0.28**	0.13**	0.09	-0.22**	0.35**	1	

Social support (friends)	0.31**	0.11*	0.11*	-0.20*	0.34**	0.63**	1

* Correlation is significant at the 0.05 level.

** Correlation is significant at the 0.01 level.

Logistic regression was used to identify variables that contribute to inadequate FV consumption. The results showed that knowledge (OR = 0.59, 95% CI = 0.39-0.88), perceived benefits (OR = 0.92, 95% CI = 0.88-0.96) and barriers (OR = 1.08, 95% CI = 1.04-1.14), self-efficacy (OR = 0.89, 95% CI = 0.83-0.95), and support from family (OR = 0.91, 95% CI = 0.83-0.99) were significant predictors of FV consumption, while age, gender, education, marital status, economic status, employment, chronic diseases, and support from friends were not. The results are presented in Table 4.

**Table 4:** Odds ratios and 95% CI obtained from logistic regression analysis for inadequate FV consumption per day

	OR (95% CI)	P
**Age**	1.06 (0.98-1.15)	0.14

**Sex**		

Male	1.0 (ref.)	

Female	0.91 (0.21-3.94)	0.89

**Education**		

Literate	1.0 (ref.)	

Illiterate	1.58 (0.74-3.38)	0.23

**Marital Status**		

Married	1.0 (ref.)	

Widowed/divorced	1.01 (0.49-2.06)	0.96

**Economic Status**		

High	1.0 (ref.)	

Intermediate	1.17 (0.44-3.10)	0.75

Low	1.38 (0.62-3.07)	0.42

**Employment Status**		

Employed	1.0 (ref.)	

Housewife	1.25 (0.275-.76)	0.76

Retired	0.72 (0.18-2.78)	0.63

**Chronic Disease**		

No	1.0 (ref.)	

Yes	1.46 (0.71-3.01)	0.29

**Knowledge**	0.59 (0.39-0.88)	0.01

**Perceived benefits**	0.92 (0.88-0.96)	< 0.001

**Perceived barriers**	1.08 (1.04-1.14)	< 0.001

**Self efficacy**	0.89 (0.83-0.95)	0.001

**Social support from family**	0.91 (0.83-0.99)	0.03

**Social support from friend**	0.97 (0.88-1.06)	0.50

## Discussion

This study revealed that FV consumption among elderly Iranians is much lower than the daily consumption recommended by WHO [34]. Furthermore, the prevalence of low FV consumption tended to increase with age. Similar studies from high-income countries such as the U.S.A. and France have shown that the prevalence of low FV consumption increases with age [35,36]. Many developing countries have no data on FV consumption patterns by their populations [37], so it seems impossible to compare our findings with countries with similar conditions.

Although previous studies [38,39] have shown significant differences between males and females in FV intake, the present study revealed no significant difference between genders. We speculate that this might be due to cultural differences among nations; or simply because most women in this study were housewives, one might argue that they had relatively adequate FV in their daily dietary intakes so males and females did not differ significantly.

As expected, this study showed that participants who were more educated (people with higher educational level) and wealthier consumed more FV. These results are similar to those reported by other investigators [40,41]. It seems that more research is needed to assess the relationship between educational level in elderly individuals and their knowledge regarding FV, to ascertain whether having higher education leads to consumption of adequate FV, or whether knowledge about FV is sufficient in itself to result in adequate FV intake regardless of educational level.

Although this study showed that most participants had good knowledge regarding the different health benefits of FV and also believed that vitamin pills are not real substitutes for fresh FV, very few participants knew about the daily amount of FV consumption or the correct size of an FV serving as recommended by WHO. Van Duyn and co-workers indicated that awareness of how many FV portions a person should eat per day is associated with higher consumption [42].

The current results provide further support to studies indicating that FV prices are a barrier to consumption by low-income consumers, so developing public policies to make FV more affordable for low-income families should be encouraged [43]. A previous study indicated that a one percent decrease in the price of FV would lead to a 2% increase in the participants' consumption of FV, and a 1% increase in family income would increase FV consumption up to 4% [44].

The findings of this study indicated that married participants consumed more FV than the widowed and divorced. A study showed that married men consumed more FV than single men but there were no differences among women in this regard [45]. This might suggest a need for further investigation on this issue or it might be necessary to study the relationship between marital status, family support and FV consumption in different cultures. However, the current findings also indicated that social support could increase FV intake among elderly individuals. Others have also reported a positive relationship between social support from family and friends and FV intake [46]. Thrasuer et al. examined types rather than sources of support as determinants of healthy eating among African American adults. They found that informational and instrumental support was associated with healthy eating [47]. In Iran, most elderly people live with members of their families and are well supported in informational, emotional and instrumental terms, and family members are responsible for the needs of those who live alone. Also, peer support predominates in providing emotional and informational support backing for them. This situation could lead to more healthy behaviors (such as FV consumption).

Participants in more advanced stages of change of FV consumption behavior were more likely to consume these foods. This is consistent with findings by other researchers [48]. These results imply that stage of readiness to change eating habits should be considered as an influencing factor while interventions for increasing FV are being planned. For example, motivational strategies for encouraging FV consumption may be more effective for elderly individuals who are in the pre-contemplation or contemplation stages, while strategies supporting the maintenance of a level of FV consumption may be more appropriate for those in the maintenance stage. In addition, there are relationships between TTM variables (benefit, barriers and self-efficacy) and stages of change regarding FV consumption. Our findings showed that those in the later stages had higher perceived benefits and self-efficacy and lower perceived barriers. Similarly, Di Noia et al. reported that individuals who had higher pros and self-efficacy were more frequently in the preparation, action, and maintenance stages than in the pre-contemplation and contemplation stages [48]. As this study showed, variables such as barriers and self-efficacy are significant predictors of FV consumption. Self-efficacy and perceived barriers are important predicting factors for diet and many other health behaviors [49,50]. Therefore, it may be suggested that improving individuals' abilities through continued education and training may lead to enhanced intake of FV. It seems important that policy makers and all who are responsible for people's health should be aware of these influencing variables.

Consistent with other previous findings [51], our study showed that elderly individuals in the normal BMI range consumed more FV. This relationship might be explained by the lower energy density and higher volume of fiber and water content in FV [52], which leads to a more ideal weight.

Given that all our respondents were members of elderly centers, the findings of this study might not be generalized to all elderly Tehran residents. These elderly might differ from others in terms of socioeconomic status, family cohesiveness, social support, and availability and access to FV. Further studies are needed to examine the mediating factors affecting FV consumption in a larger and more diverse group of elderly in Iran. In addition, it should be noted that our findings on FV intake were based on self-reported information and thus might be associated with measurement errors. Seasonal aspects were not investigated in this study; since season might influence the availability of FV, it is recommended that this be considered in future studies.

## Conclusion

The findings demonstrated that FV consumption among elderly Iranian individuals was low and varied greatly with age, education and income level. In addition, the results indicated that low perceived benefits, low self-efficacy, and perceived barriers could lead to lower consumption of FV. Therefore, it seems that in order to improve FV consumption among elderly Iranians, raising awareness, improving perception benefits and enhancing self-efficacy regarding FV consumption should receive more attention. Indeed, it is essential to plan health education programs and nutritional interventions for this group of the population.

## List of abbreviations

FV: Fruit and vegetable; CHD: Coronary heart disease; CVD: Cardiovascular disease; BMI: Body Mass Index; TTM: Transtheoretical Model; OR: odds ration.

## Competing interests

The authors declare that they have no competing interests.

## Authors' contributions

**LS **was the main investigator, analyzed the data and involved in drafting the manuscript. **HE **supervised the study, and contributed to the study design. **KM **contributed to the study design, performed the statistical analysis, and supervised the study. **SST **involved in drafting, and revising it critically for important intellectual content. **AJ **helped in writing process. **AM **contributed to the analysis, edited the paper and provided the final version. All authors read and approved the final manuscript.
